# Detection of Immune Microenvironment Changes and Immune-Related Regulators in Langerhans Cell Histiocytosis Bone Metastasis

**DOI:** 10.1155/2023/1447435

**Published:** 2023-01-19

**Authors:** Jinding Lin, Haifeng Tang, Zhitong Xu, Rongdong Zeng

**Affiliations:** Department of Orthopedics, Fujian Medical University Affiliated First Quanzhou Hospital, 362000 Quanzhou, China

## Abstract

The inflammation/immune response pathway is considered a key contributor to the development of Langerhans cell histiocytosis (LCH) bone metastasis. However, the dynamic changes in the immune microenvironment of LCH bone metastasis and critical regulators are still unclear. Expression profiling by arrays of GSE16395, GSE35340, and GSE122476 was applied to detect the immune microenvironment changes in the development of LCH bone metastasis. The single-cell high-throughput sequencing of GSE133704, involved in LCH bone lesions, was analyzed. The online database Metascape and gene set variation analysis (GSVA) algorithms were used to detect the gene function of Gene Ontology (GO) and Kyoto Encyclopedia of Genes and Genomes (KEGG). The protein–protein interaction (PPI) network of hub regulators was constructed by the STRING database. In these results, key immune cells, such as Tem cells, NK T cells, CD8(+) T cells, and Th1 cells, were identified in LCH bone metastasis. These genes, which include LAG3, TSPAN5, LPAR5, VEGFA, CXCL16, CD74, and MARCKS, may significantly correlate with the cellular infiltration of B cells, aDCs, pDCs, cytotoxic cells, T cells, CD8+ T cells, T helper cells, and Tcm cells. In conclusion, our study constructed an atlas of the immune microenvironment of LCH bone metastasis. Genes including LAG3, TSPAN5, LPAR5, VEGFA, CXCL16, CD74, and MARCKS may be involved in the development of LCH bone metastasis. The hub gene-immune cell interactive map may be a potential prognostic biomarker for the progression of LCH bone metastasis and synergetic targets for immunotherapy in LCH patients.

## 1. Introduction

Langerhans cell histiocytosis (LCH) is an inflammatory myeloid neoplasm of mixed cellularity that is clinically heterogeneous, ranging from self-resolving skin or single bone lesions to systemic forms involving the bone marrow, liver, and/or spleen [1]. The pathological features of LCH include a large number of Langerhans cells, hyperplasia, infiltration, and granuloma formation, accompanied by inflammatory cell infiltration, resulting in tissue destruction and tissue and organ dysfunction [2]. Both adults and children are susceptible to the disease, but it tends to be more prevalent in children [3, 4].

Evidence has shown that mutations of B-raf protooncogene-serine/threonine kinase (BRAF) in the mitogen-activated protein kinase pathway play important roles in the progression of LCH, although its pathogenesis is not fully understood [5]. Recently, activating somatic mutations in mitogen-activated protein kinase (MAPK) pathway genes, most notably BRAFV600E, have been discovered in almost all cases of LCH. Moreover, Bigenwald et al. also reported that enforced expression of the BRAF V600E mutation in early mouse and human multipotent hematopoietic progenitor cells induced a senescence program that led to hematopoietic progenitor cell growth arrest, apoptosis resistance, and senescence-associated secretory phenotype, which contribute to the formation of LCH lesions [6]. In addition, other members in the MAPK pathway, such as MAP2K1 and ARAF, were also activated due to mutation, which could contribute to LCH. However, the in-depth exploration of the MAPK pathway has not brought qualitative improvement to the treatment of LCH [7, 8]. We still need to find more diversified targets for systematic treatment.

It is generally believed that abnormalities of the immune microenvironment often exist in LCH lesions. Evidence concerning LCH immunology suggests that clonal changes in dendritic cells (DCs) might underlie the aberrant immune interaction with T cells, leading to a unique pathological picture that combines features of carcinogenesis and chronic inflammation [9]. Moreover, DC-derived osteoclasts may be directly involved in forms of Langerhans cell histiocytosis, characterized by the accumulation of immature skin DCs and chronic lytic bone lesions [10]. In addition, for T cells, intralesional CD8+ T cells showed blunted expression of Tc1/Tc2 cytokines and impaired effector function, while regulatory T cells demonstrated intact suppressive activity [11]. However, the mechanisms that lead to disturbances in the immune microenvironment also need to be further studied.

The development of single-cell sequencing technology has brought new ideas to solve this problem. Halbritter et al. launched a single-cell analysis of LCH and uncovered an unexpected degree of cellular, transcriptomic, and epigenomic heterogeneity among LCH cells, indicative of complex developmental hierarchies in LCH lesions [12]. Moreover, Shi et al. analyzed the differences in the phenotypes of peripheral immune cells and MAPK pathway expression of different subsets of cells in children with LCH by single-cell sequencing and found that the decreased frequency of plasmacytoid dendritic cells was significantly correlated with the severity of the disease, which might contribute to the improvement of clinical diagnostics and therapeutics and aid in the development of personalized medicine approaches [13].

However, the mechanism by which different hub genes, pathways, and immune cells interact with each other, thus leading to changes in the immune microenvironment and inducing diseases, is still unknown. Therefore, based on the public database and bioinformatics analysis, this study was proposed to analyze different phenotypes of key LCH genes, pathways, and contacts between the immune microenvironment and different molecular phenotypic differences in the LCH immune microenvironment to further study the immunological mechanism of LCH and potential targets for the accuracy of LCH system treatment.

## 2. Methods

### 2.1. Data Processing and DEG Mining

Expression profiling by arrays from GSE16395 [14], GSE35340 [15], and GSE122476 [16] was downloaded from GEO (http://www.ncbi.nlm.nih.gov/geo/) [17], expression profiling arrays which were generated using the GPL570 (HG-U133_Plus_2) Affymetrix Human Genome U133 Plus 2.0 Array (Affymetrix, Santa Clara, CA) and GPL17586 Affymetrix Human Transcriptome Array 2.0 (Affymetrix, Santa Clara, CA). All the samples of GSE16395, including LCH (CD207(+), CD3(+), and control samples), were selected to explore the immunomodulation atlas of LCH lesions. Meanwhile, the GSE35340 and GSE122476 datasets were included to validate the immunomodulation changes. In addition, single-cell high-throughput sequencing of GSE133704 [12] was also downloaded for future analysis. The process of data preprocessing was based on Zou et al. [18, 19]. The Benjamini-Hochberg method was used to adjust the original *p* values, and the false discovery rate (FDR) procedure was used to calculate fold-change (FC). Gene expression values of |log2 FC| > 1 and adjusted *p* < 0.05 were used for filtering DEGs.

### 2.2. Immune Cell Infiltration Analysis

To compare the differences in the immune microenvironment of different molecular phenotypes of LCH, we performed immune infiltration analysis on the downloaded GSE16395 dataset based on CIBERSORT (https://cibersortx.stanford.edu/) [19, 20]. After the gene expression matrix of GSE16395 containing all types of LCH, CIBERSORT was run with the following options: relative and absolute modes together, LM24 signature gene file, 100 permutations, and quantile normalization disabled [19]. After the generation of the immune microenvironment matrix of each sample, we further analyzed the immune microenvironment of LCH of each cell phenotype.

### 2.3. Pseudotime Analysis of Highly Variable Features in Single-Cell Sequencing

To evaluate the sequence and trajectory of cell-to-cell transformation and succession in LCH with different molecular phenotypes, highly variable feature (HVF) mining and pseudotime analysis were used in GSE133704 based on R [12]. For HVF, after normalization, genes that expressed significant differences in samples were identified. The detailed method of pseudotime analysis can be found in Hafemeister and Satija's work [21]. First, after data normalization, the feature genes that defined a cell's process were chosen. Then, data dimensionality was reduced, and pseudotime marker gene sets were obtained. The root state parameter was utilized to specify the starting end. The branch that contained the most cells at state 0 was then identified.

### 2.4. Functional Enrichment Analysis and Protein–Protein Interaction

First, to estimate the variation in pathway activity among samples with different phenotypes in GSE16359, gene set variation analysis (GSVA) [22], a nonparametric unsupervised method, was used based on the gene expression matrix. Then, to clarify the potential biological functions involved in hub genes such as co-DEGs in different phenotypes and pseudotime marker genes, Gene Ontology (GO) and Kyoto Encyclopedia of Genes and Genomes (KEGG) enrichment analyses were carried out in the online database Metascape (https://metascape.org/), and *p* values < 0.01, minimum counts of 3, and enrichment factors > 1.5 were regarded as significant [23]. Protein–protein interactions concerning hub genes were also enriched in Metascape.

### 2.5. Partial Correlation Analysis

To exclude the interference of other covariables, we used partial correlation analysis (PCA) to analyze the correlation between hub genes and immune cells in different datasets [24]. The correlations of infiltrating immune cells were determined via the following guide for the value of partial cor: 0.00–0.19: “very weak,” 0.20–0.39: “weak,” 0.40–0.59: “moderate,” 0.60–0.79: “strong,” and 0.80–1.0: “very strong.”

## 3. Results

### 3.1. LCH with Different Molecular Phenotypes Had Different Immune Microenvironments

**Figure 1:**
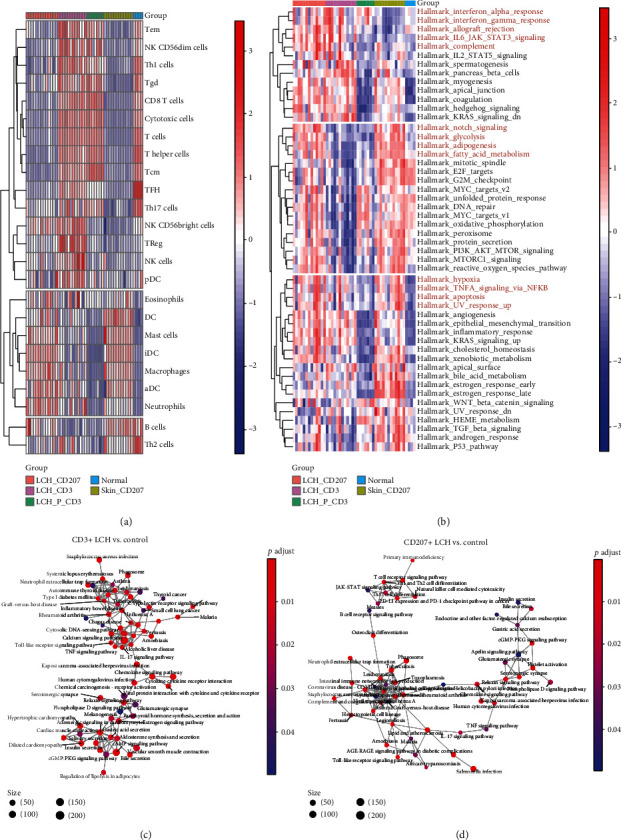
Immune infiltration subtypes and pathway interactive network detection. (a) The burden of infiltrating immune cells among the different Langerhans cell histiocytosis (LCH) subtypes was identified based on the ssGSEA algorithm with system-level gene expression data. (b) Hierarchical clustering analysis showed that the immune/inflammation response, hypoxia, and metabolic dysfunction were significantly detected. (c, d) The pathway interactive networks were identified in the comparison of CD3+ LCH vs control and CD207+ LCH vs control.

After selection, all samples of GSE16395 were enrolled in our study containing the types of LCH (CD207(+), CD3(+), and control samples). Immune cell infiltration analysis showed that immune cells such as Tem cells, NK T cells, CD8(+) T cells, and Th1 cells were significantly different among different cell phenotypes (Figure 1(a)), indicating that different phenotypes of LCH had different immune microenvironments. Moreover, GSVA and pathway enrichment analysis also showed that they had different signaling pathways among different groups (Figures 1(b)–1(d)).

### 3.2. Functional Enrichment Analysis of Marker Genes in Pseudotime Clusters

**Figure 2:**
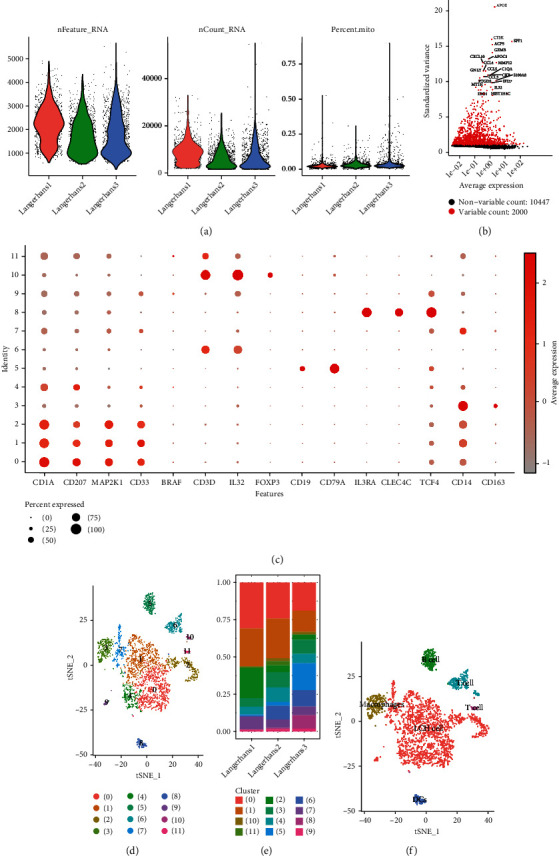
Langerhans cell histiocytosis (LCH) single-cell sequencing analysis. (a) The box plot shows the LCH sample feature count and mitochondrial content. (b) The dot plot presents the variable features in scRNA analysis. (c) The hub immune marker expression levels are presented in different cell clusters. (d–f) The spatial distribution (d) and annotation (f) of LCH single-cell clustering were detected via singleR analysis, as well as the percentage of the cell-cluster ratio (e).

After quality control, all 7 samples from GES133704 were used for single-cell sequencing analysis (Figure 2(a)). 2000 HVF were found (Figures 2(b) and 2(c)). After t-SNE, 11 clusters were found, and those in the middle were related to LCH (Figures 2(d)–2(f)).

**Figure 3:**
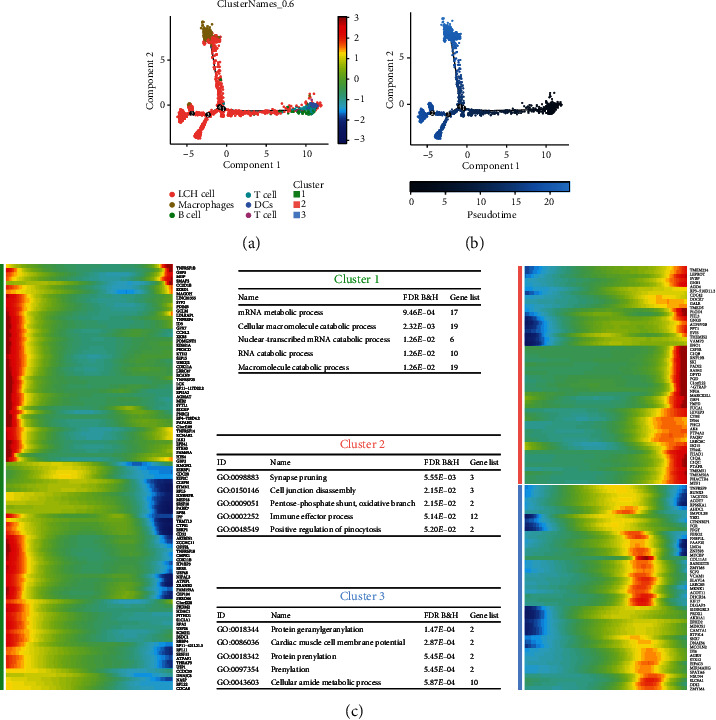
Pseudotime analysis in LCH single-cell sequencing. (a, b) The DDTree plots are presented and show the annotated cell and station development. (c) Variable features were identified in different cell states, and gene function enrichment analysis was also applied.

After pseudotime analysis, 3 clusters were enriched (Figures 3(a) and 3(b)). We analyzed the enrichment of HVF in these three clusters, which were involved in different biological processes (Figure 3(c)). The first cluster was involved in mRNA metabolic process (FDR = 9.46*E* − 04), cellular macromolecule catabolic process (FDR = 2.32*E* − 03), nuclear-transcribed mRNA catabolic process (FDR = 1.26*E* − 02), and so on, while Cluster 2 was involved in synapse pruning (FDR = 5.55*E* − 03) and cell junction disassembly (FDR = 2.15*E* − 02), and Cluster 3 was involved in protein geranylgeranylation (FDR = 1.47*E* − 04) and cardiac muscle cell membrane potential (FDR = 2.87*E* − 04).

### 3.3. Co-DEGs Play Roles in LCH via Different Pathways

**Figure 4:**
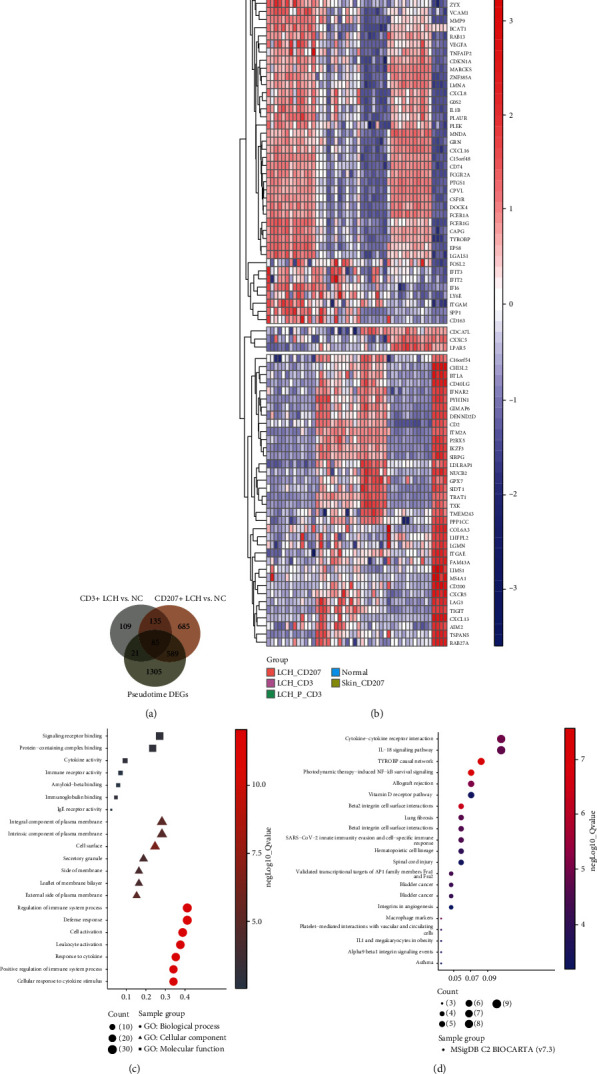
LCH bone metastasis-related hub regulator detection. (a) The Venn plot shows the hub markers among the comparison of CD3+ LCH vs control, CD207+ LCH vs control, and scRNA pseudotime differentially expressed genes. (b) The hierarchical clustering heat map shows the 85 hub gene expression levels in different LCH subtypes. (c, d) The biological process, cellular component, and molecular function enrichment results are shown in (c), and the BIOCARTA pathway enrichment is shown in (d).

After mining GSE16395 and GSE133704, we found that 85 of the hub genes were significant in both the pseudotime analysis and two cell phenotypes (Figure 4(a)).  Figure 4(b) illustrates the expression value of all 85 genes. Through gene enrichment analysis, we found that these 85 genes participate in the regulation of multiple signaling pathways, and most of them are related to immune cells and inflammatory responses (Figures 4(c) and 4(d)).

### 3.4. Hub Genes Involved in the Immune Microenvironment in Different Phenotypes of LCH

**Figure 5:**
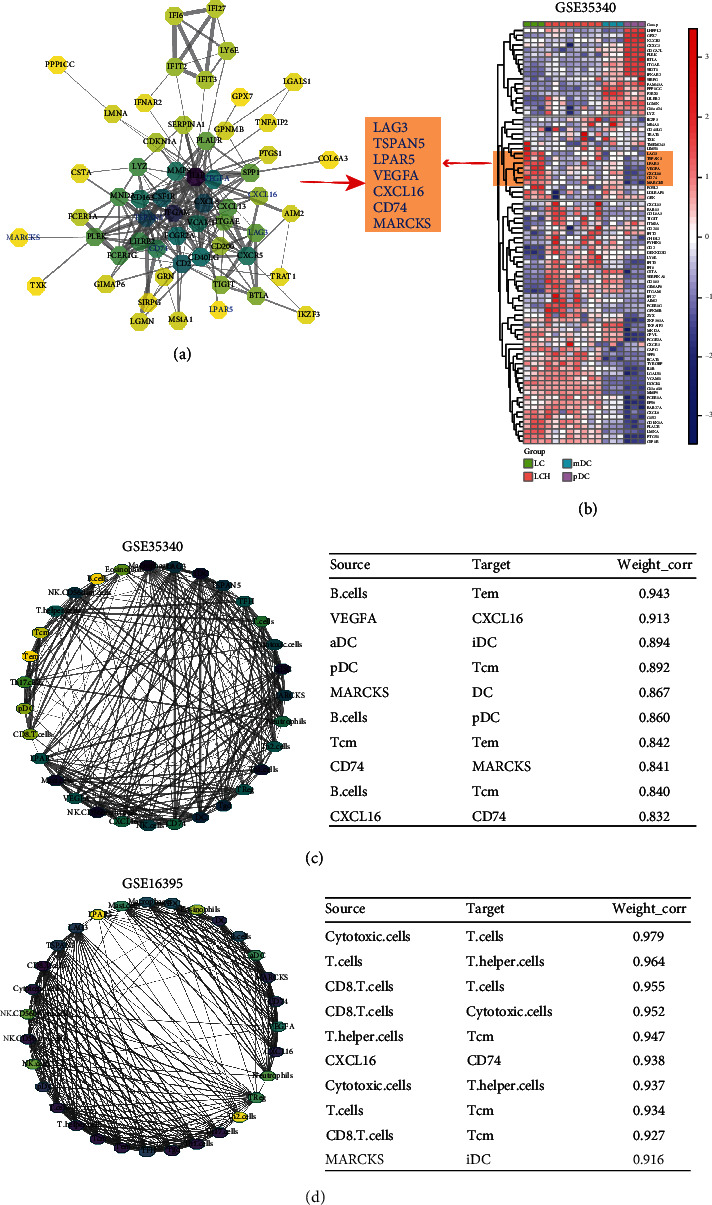
LCH bone metastasis-related hub gene-immune cell interactive network detection. (a) After submitting the 85 bone metastasis-related hub genes to the STRING database, based on seven evidence channels and cut-off by the highest confidence score, the interactive pairs were obtained. The PPI network can be abstracted as a graph consisting of a node set and edge set, where the nodes represent the proteins of the network and each edge represents the pairwise protein interaction. Subsequently, by calculating the node's degree, the 7 hub genes were considered the central regulators. (b) The hierarchical clustering analysis showed a lower expression level in the external dataset of GSE35340. (c, d) The green-cell interactive network presented a close correlation between the expression levels of 7 hub markers and the relative infiltration value of immune cells.

In GSE35340, we found 82 significant genes in 4 groups (Figure 5(b)), and the interactive network showed that some of these genes were hub genes, including LAG3, TSPAN5, LPAR5, VEGFA, CXCL16, CD74, and MARCKS (Figure 5(a)). Interestingly, these hub genes were also significant in GSE35340 and GSE16395, and partial correlation analysis showed that these genes were correlated with immune cells with a high correlation coefficient (Figures 5(c) and 5(d)). Furthermore, there are strong correlations between hub genes and immune cells and between immune cells and other types of immune cells, suggesting that gene-immune cell and immune cell-immune cell interactions are involved in different mechanisms of LCH of different cell phenotypes.

## 4. Discussion

In this study, we analyzed the differences in hub genes, pathways, and immune microenvironment components of different molecular phenotypes of LCH using multiple datasets and bioinformatics analysis techniques, including difference analysis, single-cell sequencing analysis, pathway enrichment analysis, and immune infiltration analysis. We found 85 co-DEGs in different phenotypes of LCH, which mainly correlated with an abnormal immune microenvironment.

Recent evidence has shown that immune indices are predictive of the severity of LCH [25]. LCH is characterized by lesions containing inflammatory immune cells, including myeloid cells and T cells, especially DCs [26]. Paredes et al. found that the levels of macrophages, mature dendritic cells, Tregs, and cytotoxic lymphocytes were significantly abnormal and that different phenotypes of LCH have different immune microenvironment abnormalities [27]. Zeng et al. also emphasized the importance of immune microenvironment changes in the process of LCH lesions. Their research demonstrated that the BRAF V600E mutation in LCH may be significantly correlated with the regulation of programmed cell death 1 ligand 1 (PDL1) expression and forkhead box protein 3 (FOXP3)(+) regulatory T-cell infiltration and closely related to the long-term survival of patients [28].

In our research, similarly, we found that these immune cells, including effective memory T cells (Tem), central memory T cells (Tcm), Th cells, Tregs, and many other immune cells, were abnormal in LCH and that the phenotypes of LCH have different abundances of immune cells. Although the mechanism of these cells, such as Tregs, has not been clarified [29], we found that various cells may interact with each other to jointly regulate the progression of LCH through analysis.

Evidence has shown that mutations in members of the MAPK pathway contribute to LCH via various mechanisms, including several immune-related signaling pathways [13]. Meanwhile, in our study, we also found that hub genes represented by members of the MAPK pathway are involved in multiple inflammatory signaling pathways (such as leukocyte activation, response to cytokines, and cytokine−cytokine receptor interaction), and these hub genes have common abnormal changes in LCH of different cell phenotypes and are related to abnormal infiltration of immune cells.

In addition, we also found that myristoylated alanine-rich protein kinase C substrate (MARCKS), a member of the MAPK family and a co-DEG among arrays and single-cell sequences, is highly related to signaling pathways and an abnormal immune microenvironment. MARCKS is a ubiquitous, highly conserved membrane-associated protein involved in the structural modulation of the actin cytoskeleton, chemotaxis, motility, cell adhesion, phagocytosis, and exocytosis, being expressed mostly in innate immune cells and promoting the inflammation-driven migration and adhesion of cells and the secretion of cytokines [30]. MARCKS can activate multiple pathways, including NF-kappa B, to promote tumorigenesis and development [31]. Meanwhile, evidence has shown that MARCKS may influence M2 polarization and immune escape and is associated with poor prognosis and immune cell infiltration in tumors [32]. However, there is little literature on the role of this gene in LCH. In our research, by enrichment analysis and partial correlation analysis, we found that MARCKS was highly correlated with hub genes such as CD74 and immune cells such as DCs, which are responsible for LCH. Since MARCKS is currently viewed as a potential target for immunotherapy and chemosensitivity, it is reasonable to believe that it will also be a potential therapeutic target for LCH [33, 34].

However, our research is an integrated analysis of scRNA and transcriptome microarray datasets in LCH, which belongs to systematic analysis and inductive research based on public databases. Although the pathological mechanism of LCH systemic damage and bone invasion has been elaborated to a certain extent, much work, including clinical tissues, phenotypic association analysis, and molecular experiments in vitro and in vivo, is still needed to verify the specific molecular mechanism. In addition, we also cannot explain the translational value of relevant key regulators for prognosis prediction and clinical treatment of immunologically related LCH lesions. In the future, we will apply for corresponding ethical consent and collect LCH pathological tissues for in-depth research. Further exploration of the disease will be performed with the help of the findings of this manuscript.

## Data Availability

The data and analysis scripts that support the findings of this study are available from the corresponding author upon reasonable request.
